# Amelioration of obesity-related biomarkers by *Lactobacillus sakei* CJLS03 in a high-fat diet-induced obese murine model

**DOI:** 10.1038/s41598-019-43092-y

**Published:** 2019-05-02

**Authors:** Yosep Ji, Soyoung Park, Youngmee Chung, Bobae Kim, Haryung Park, Eunchong Huang, Dahye Jeong, Hoe-Yune Jung, Bongjoon Kim, Chang-Kee Hyun, Wilhelm H. Holzapfel

**Affiliations:** 1 Advanced Green Energy and Environment, Handong Global University, Pohang, Gyungbuk 37554 Republic of Korea; 2 CJ Blossom Park, 42, Gwanggyo-ro, Yeongtong-gu, Suwon-si, Gyeonggi-do, 16495 Republic of Korea; 3 Department of Life Science, Division of Integrative Biosciences and Biotechnology, POSTECH, Pohang, Gyungbuk 37673 Republic of Korea; 4 School of Life Science, Handong Global University, Pohang, Gyungbuk 37554 Republic of Korea

**Keywords:** Biochemistry, Applied microbiology

## Abstract

Recent progresses in clinical diagnostic analyses have demonstrated the decisive influence of host gut microbiota on the status of metabolic disorders. Short chain fatty acids (SCFAs) produced by gut microbiota, in particular, are considered as a key biomarker, both of communication between gut microbiota and the host, and of impact on host metabolic homeostasis. Microbiota modulation and concomitant anti-obesity effects of probiotics have been reported by different researchers. However, the underlying modulatory functions of probiotics on gut microbiota towards host metabolic homeostasis are still not fully understood. In this study, the impact of *Lactobacillus sakei* CJLS03 (isolated from Korean kimchi) on obesity-related biomarkers was investigated using a diet-induced obese mouse model. Body weight increase, SCFAs, the gut microbiota and various obesity-associated biomarkers were significantly and beneficially influenced by *L. sakei* CJLS03 administration compared to the control groups. Analytical data on faecal samples support the role of the colonic microbial population in SCFA production. The composition of the latter may be influenced by modulation of the distal gastro-intestinal microbiota by putative probiotics such as *L. sakei* CJLS03.

## Introduction

Obesity has become a major social issue associated with health and quality of life. Approximately one-third of the world’s population is reported as obese or overweight, and this phenomenon is expected to increase further^1,2^. Currently, several drugs are being used under approval of Food and Drug Administration (FDA) around world for the purpose of weight loss with known mechanisms that promote fat metabolism, inhibit absorption of consumed fat, or suppress appetite. However, such pharmacological approach may cause serious side effects, resulting in a problem in continuous administration^3^. Accordingly, it is necessary to consider a new therapeutic approach that is safer and improves the harmony of the overall metabolism in the body.

Probiotics are defined as “live microorganisms that, when administered in adequate amounts, confer beneficial effects on the host”^4^. Combinations of antibiotics, probiotics and prebiotics have been suggested as a basis for novel therapeutic approaches for the beneficial modulation of commensal microbiota^4^. Proven probiotics have a relatively recent history of safe use and reported beneficial effects. Various papers have reported that the resulting beneficial effects include amelioration of obesity and of associated immuno-metabolic diseases^4–8^. Most of the studies (both *in vivo* and clinical trials) reported that consumption of probiotics resulted in reduced fat accumulation and relief in the level of biomarkers related to metabolic disorders such as blood glucose and triglycerides. Administration of *L. sakei* strain OK67 isolated from kimchi ameliorated HFD induced body and epididymal fat weight gains and reduced expression of various pro-inflammatory cytokines and serum endotoxin in a HFD induced obesity mouse model^9^. An earlier study has reported on the reduction of body weight, body mass index and visceral and subcutaneous fat by application of *L. gasseri* SBT2055 (in fermented milk) in a double-blind randomized, placebo-controlled trial over 12 weeks^10^. Despite the potent beneficial effects against metabolic syndrome, the mechanisms underlying these functional probiotic attributes are thus far only partially understood^11^. Novel insights in the complex relationship between gut microbiota and host health suggest that beneficial effects may be linked to the ‘normalisation’ of the host gut microbiota^1,11^. Thus, an imbalance (dysbiosis) of intestinal microbiota may lead to obesity and related metabolic disorders^12,13^. A ‘dysbiotic’ or imbalanced condition reflects disruption and negative shifts in abundance, diversity and relative distribution of the gut microbiota, and, as for metabolic syndrome, may also be associated with inflammatory bowel diseases (IBD), cancer, neurological disorders and type 1 and type 2 diabetes^12,14–16^.

The key role of the gut microbiota in host metabolism is reflected by a higher utilization of indigestible carbohydrates in obese people^17^ for which increased numbers of indigestible carbohydrate utilizing microbiota have been reported^18^. With caecal short chain fatty acids (SCFAs) providing 10% of the daily absorbable energy, changes in the gastrointestinal tract (GIT) microbiota will strongly impact host energy metabolism^19^, as is also reflected in the higher SCFA concentrations in faecal samples of overweight and obese individuals^20^. However, an increasing number of reports describe beneficial (including anti-obesogenic) functions of SCFAs; these are confirmed by a recent study in which germ-free mice received transplanted faecal material of discordant obese and lean twins^21^. Despite the well-recognized role of SCFAs as extra energy source for *de novo* lipogenesis, accumulating literature reports explaining a new role of SCFA’s such as G protein coupled receptor 41 and 43 (GPR41/GPR43) mediated satiety and insulin sensitivity regulation^22–26^. The importance of SCFA production by commensal microbiota is also emphasised by other host health benefits such as the production of vitamins^27^.

In this study we administered *L. sakei* strain CJLS03 (isolated from Korean kimchi) to a diet-induced obese C57BL/6 murine model and analysed both faecal microbiota modulation and the SCFA level of the serum and faeces by gas chromatography (GC/FID) while the expression level of various obesity related genes was determined in the liver and epididymal adipose tissue.

## Results

### Impact of *L. sakei* CJLS03 on weight gain and obesity associated biomarkers

**Figure 1:**
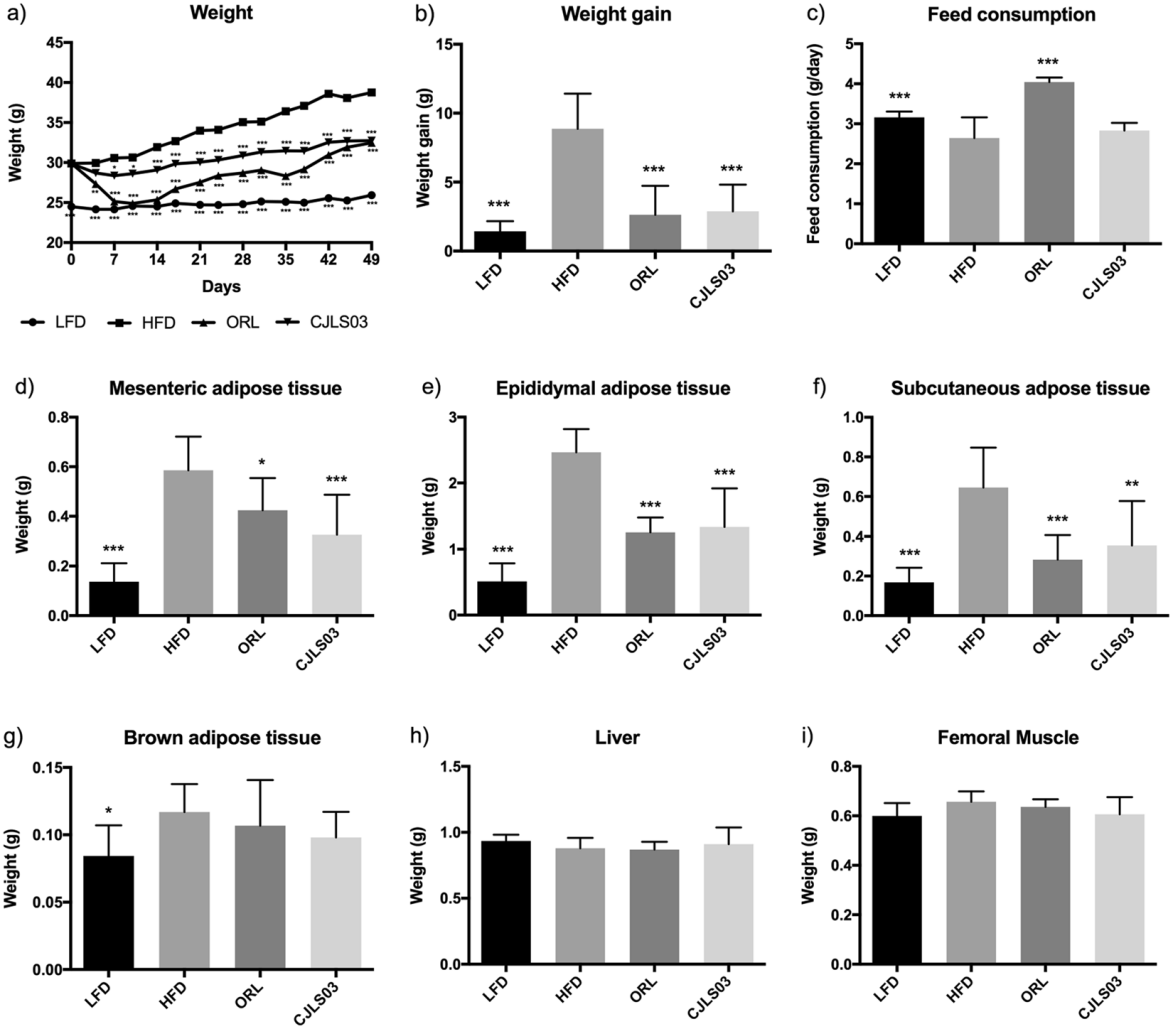
Anti-obesity effect of *L. sakei* CJLS03 determined in a diet-induced obesity (DIO) mouse model. The level of significance was measured using ANOVA, Dunnett’s multiple comparisons test was used to distinguish the level of significance based on probability of 0.05 (*), 0.01 (**) and <0.001 (***) compared to the HFD group. Feeding groups: LFD: low-fat diet plus PBS; HFD: high-fat diet plus PBS; ORL: high-fat diet plus orlistat; CJLS03: high-fat diet plus *L. sakei* CJLS03.

**Figure 2:**
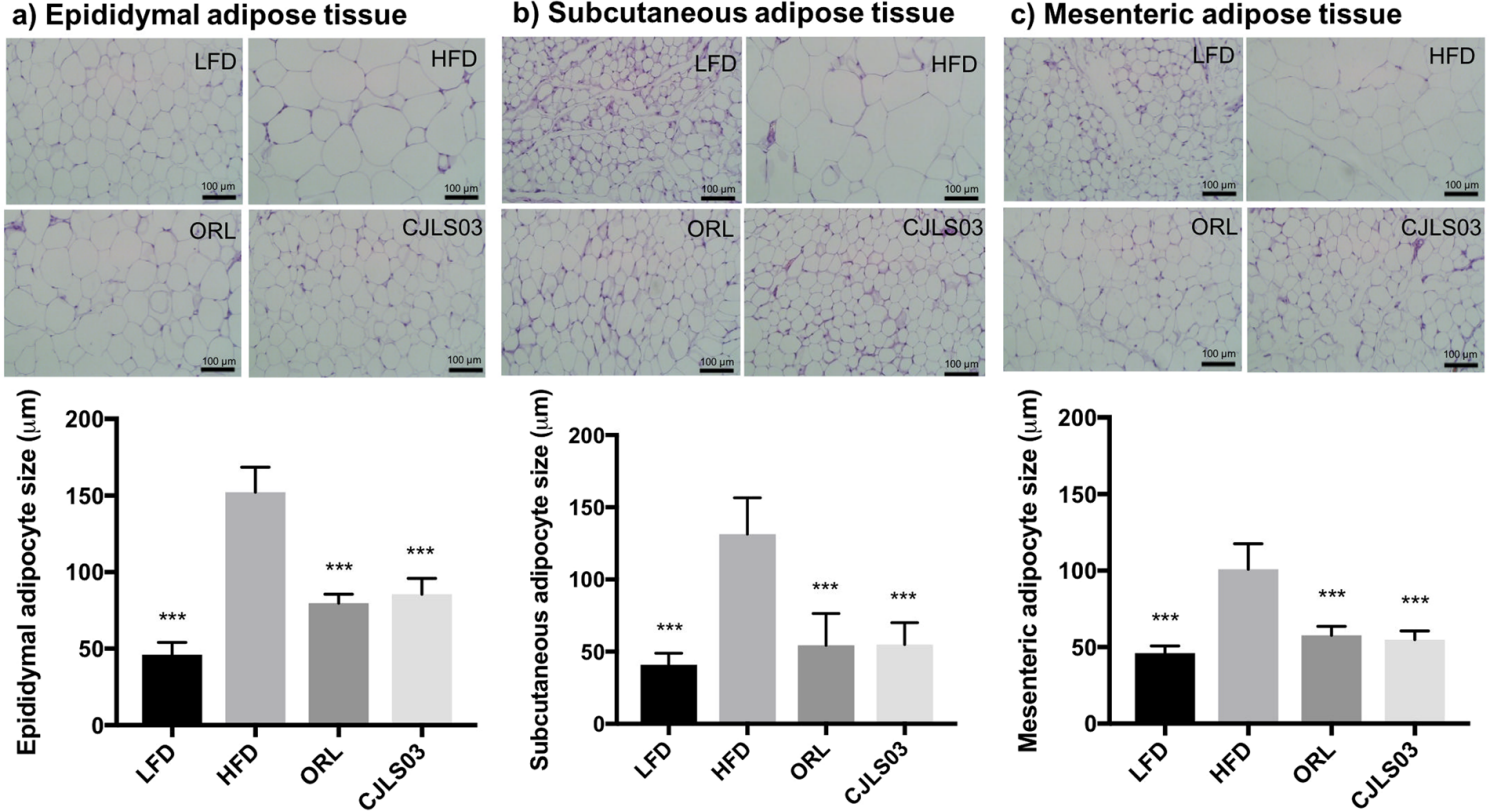
Adipocyte size of each group. ANOVA, Dunnett’s multiple comparisons test was used to distinguish the level of significance based on probability of 0.05 (*), 0.01 (**) and <0.001 (***) compared to the HFD group. Feeding groups: LFD: low-fat diet plus PBS; HFD: high-fat diet and PBS; ORL: high-fat diet plus orlistat; CJLS03 high-fat diet plus *L. sakei* CJLS03.

Our previous experiments involving three different *L. sakei* strains have shown the most promising anti-obesity effects for *L. sakei* CJLS03^28^, but prompted further research on related biomarkers. In this investigation we used orlistat as control, this drug being the commercially available anti-obesity drug in various countries such as the United States, the European Union, Australia and Japan; in some countries it is available over the counter when used at a dose of under 60 mg. It was reported to show a distinct weight loss effect with minimal systemic absorption^29,30^. Mice on a high-fat diet (HFD) showed significantly increased body weight gain and decreased feed consumption after 7 weeks compared to low-fat diet (LFD) fed controls (Fig. 1a–c). Weights of epididymal, mesenteric, subcutaneous and interscapular brown adipose tissues were markedly increased by HFD feeding, whereas weights of the liver and femoral muscle were not altered compared to the LFD control group (Fig. 1d–i). Administration of *L. sakei* CJLS03 resulted in a significantly lower weight gain of the experimental group, compared to the HFD-fed control, but with no change in feed consumption (Fig. 1a–c). The *L. sakei* CJLS03 treated group showed a significant weight reduction which was also reflected in the mesenteric, epididymal and subcutaneous adipose tissues, but without any significant reduction in the brown adipose tissue compared to the HFD control group (Fig. 1d–g). Liver and femoral muscle weight did not show any significant differences among the groups when compared to the HFD control (Fig. 1h,i). Body and adipose tissue weights were reduced, and feed consumption was significantly increased in the orlistat (ORL)-treated group compared to the HFD group. Haematoxylin and eosin microscopy showed that *L. sakei* CJLS03 supplementation significantly reduced the average size of epididymal, subcutaneous and mesenteric adipocytes in comparison to the HFD control group (Fig. 2). Subcutaneous and mesenteric adipose tissue sizes were reduced to the level of the ORL and LFD groups with *L. sakei* CJLS03 administration (Fig. 2b,c). Moreover, the *L. sakei* CJLS03 treatment was less effective than for the ORL and LFD groups in the reduction of epididymal adipose tissue (Fig. 2a).

**Figure 3:**
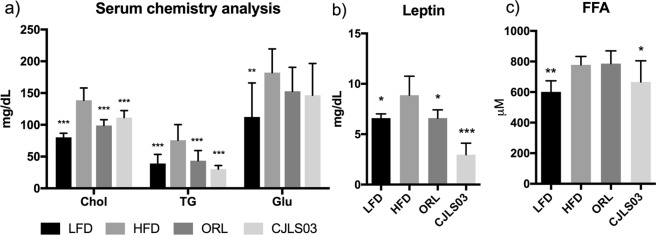
Serum biomarkers determined after administration of *L. sakei* CJLS03 in the DIO mouse model using ANOVA, Dunnett’s multiple comparisons test was used to distinguish the level of significance based on probability of 0.05 (*), 0.01 (**) and <0.001 (***) compared to the HFD group. Feeding groups: LFD: low-fat diet plus PBS; HFD: high-fat diet and PBS; ORL: high-fat diet plus orlistat; CJLS03 high-fat diet plus *L. sakei* CJLS03. FFA: Free fatty acids.

Obesity associated serum biomarkers such as cholesterol, triglycerides and glucose were analysed. Compared to the HFD group, serum cholesterol and triglycerides (TG) were significantly reduced in the *L. sakei* CJLS03 and ORL feeding groups (Fig. 3a), and (including glucose) also in the LFD control group. Serum leptin was significantly lower in the LFD, ORL and *L. sakei* CJLS03 groups while those receiving *L. sakei* CJLS03 showed the highest level of significance (Fig. 3b). Concentrations of free fatty acids (FFA) in the serum decreased significantly when *L. sakei* CJLS03 administration was compared to the HFD control group (Fig. 3c).

### Influence of *L. sakei* CJLS03 administration on biomarkers in the epididymal adipose tissue

**Figure 4:**
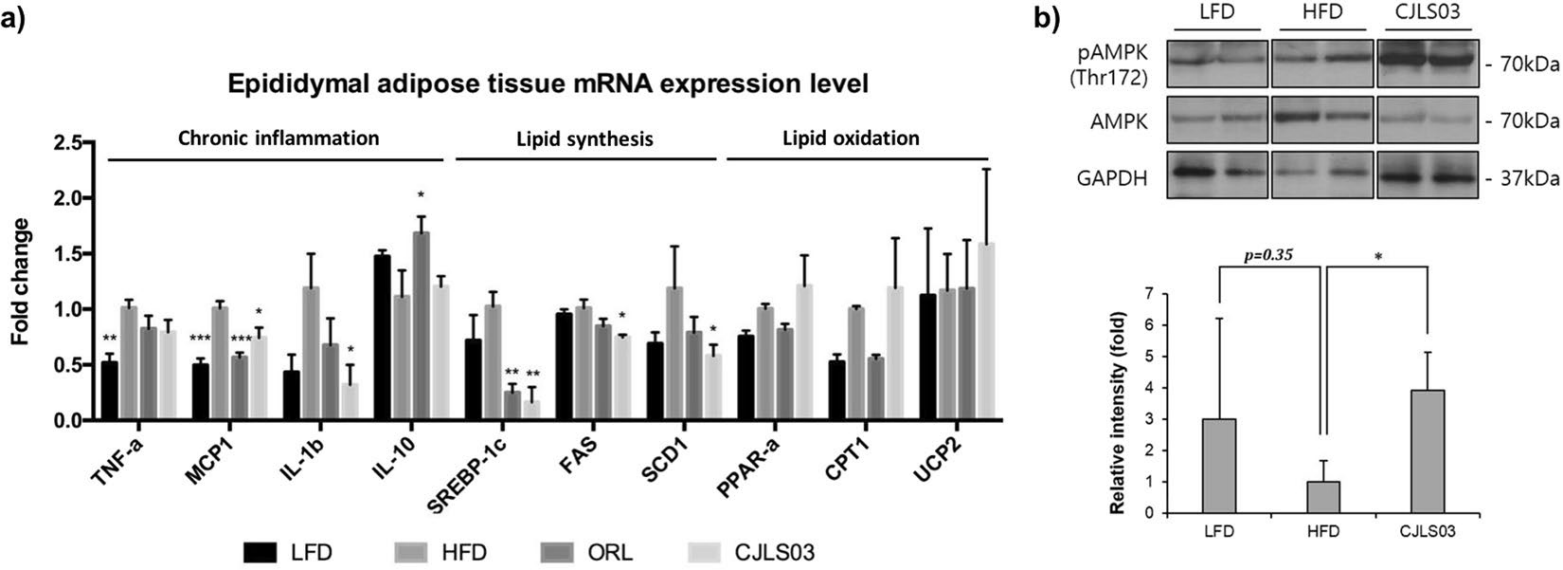
Host obesity and immune associated biomarkers in epididymal adipose tissue after *L. sakei* CJLS03 application in the DIO mouse model using (**a**) mRNA based qRT-PCR method and (**b**) Western blot. Full-length Western blots are shown Supplementary Figure S1. The mRNA expression levels and protein loadings were normalized to the expression level of glyceraldehyde 3-phosphate dehydrogenase (GAPDH). ANOVA, Dunnett’s multiple comparisons test was used to distinguish the level of significance based on probability of 0.05 (*), 0.01 (**) and <0.001 (***) compared to the HFD group. Feeding groups: LFD: low-fat diet plus PBS; HFD: high-fat diet and PBS; ORL: high-fat diet plus orlistat; CJLS03 high-fat diet plus *L. sakei* CJLS03.

Various biomarkers in the epididymal adipose tissue (EAT) were significantly influenced by *L. sakei* CJLS03 supplementation when compared to the HFD control group. Low-grade chronic inflammation in adipose tissue is one of the major causes for the development of obesity-induced insulin resistance and metabolic impairments^31^. HFD feeding resulted in significantly increased gene expression of tumour necrosis factor alpha (TNF-a) and monocyte chemotactic protein 1 (MCP1) compared to the LFD control group (Fig. 4a). The *L. sakei* CJLS03 feeding group showed significant reduction in some pro-inflammatory cytokines in the EAT, such as MCP1 and interleukin 1 beta (IL-1b) in comparison with the HFD control group. Compared to the HFD group, MCP1 was also significantly reduced in the ORL group while interleukin 10 (IL-10), an anti-inflammatory cytokine, was significantly increased (Fig. 4a). Consistent with the decrease of adipose tissue weight (Fig. 1e), the mRNA levels of fatty acid synthesis gene expression were reduced in the HFD group receiving *L. sakei* CJLS03, including the sterol regulatory element-binding protein 1 (SREBP-1c), fatty acid synthase (FAS) and stearoyl-CoA desaturase-1 (SCD1). ORL group showed significantly reduced gene expression levels of SREBP-1c compared to HFD control group. Activation of AMP-activated kinase (AMPK), a central regulator of cellular energy homeostasis, triggers catalytic processes to generate ATP while inhibiting anabolic pathways^32^. In comparison to the LFD and HFD groups, the protein level of phosphorylated AMPK was significantly increased in the *L. sakei* CJLS03 feeding group (Fig. 4b) (see also Supplementary Figure S1).

### Modulation of intestinal microbiota and short chain fatty acid composition by *L. sakei* CJLS03 treatment

**Figure 5:**
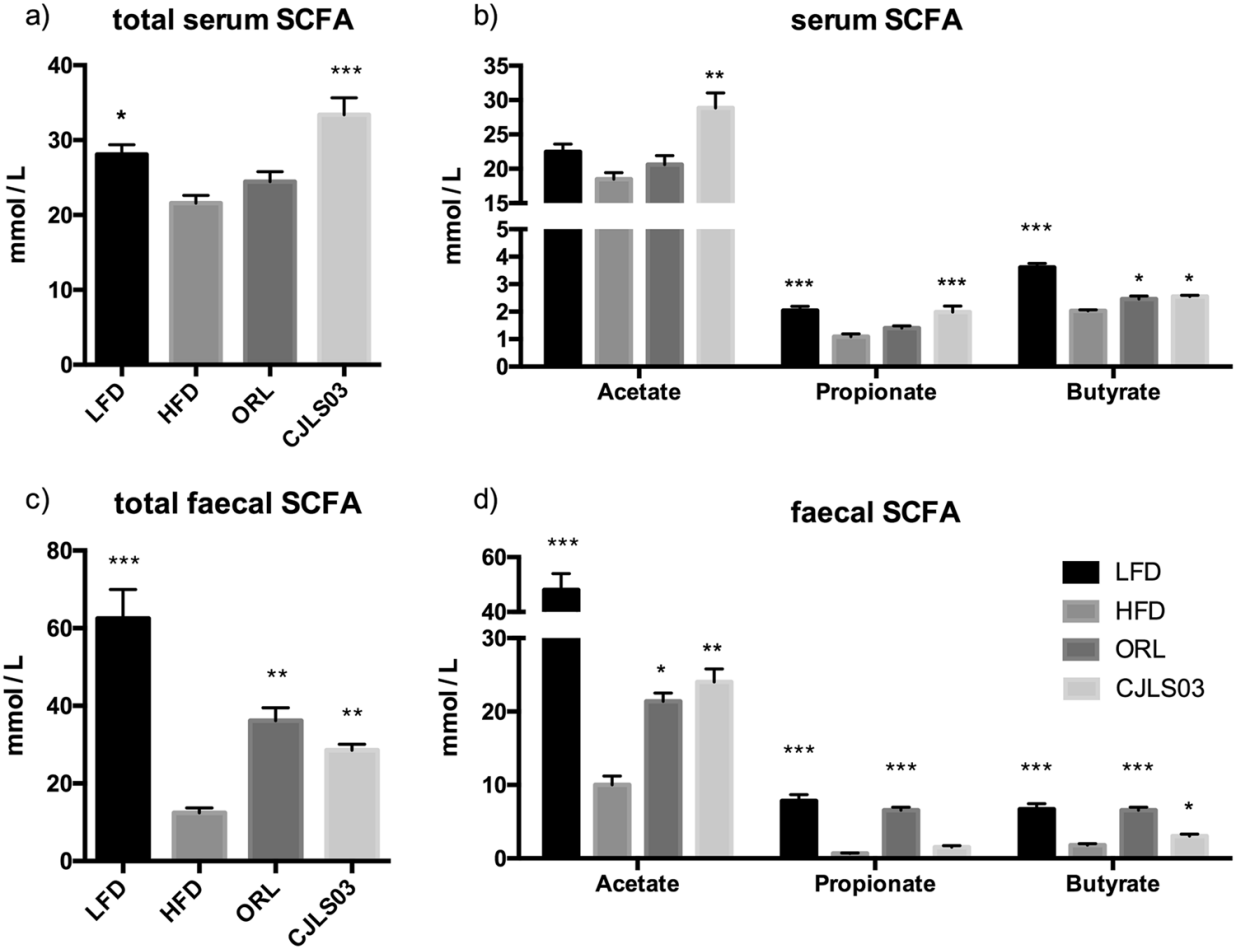
Results from gas chromatographic analysis of short chain fatty acids from serum (**a,b**) and faecal (**c,d**) samples after application of *L. sakei* CJLS03 in a DIO mouse model. ANOVA, Dunnett’s multiple comparisons test was used to distinguish the level of significance based on probability of 0.05 (*), 0.01 (**) and <0.001 (***) compared to the HFD group. Feeding groups: LFD: low-fat diet plus PBS; HFD: high-fat diet and PBS; ORL: high-fat diet plus orlistat; CJLS03 high-fat diet plus *L. sakei* CJLS03.

**Figure 6:**
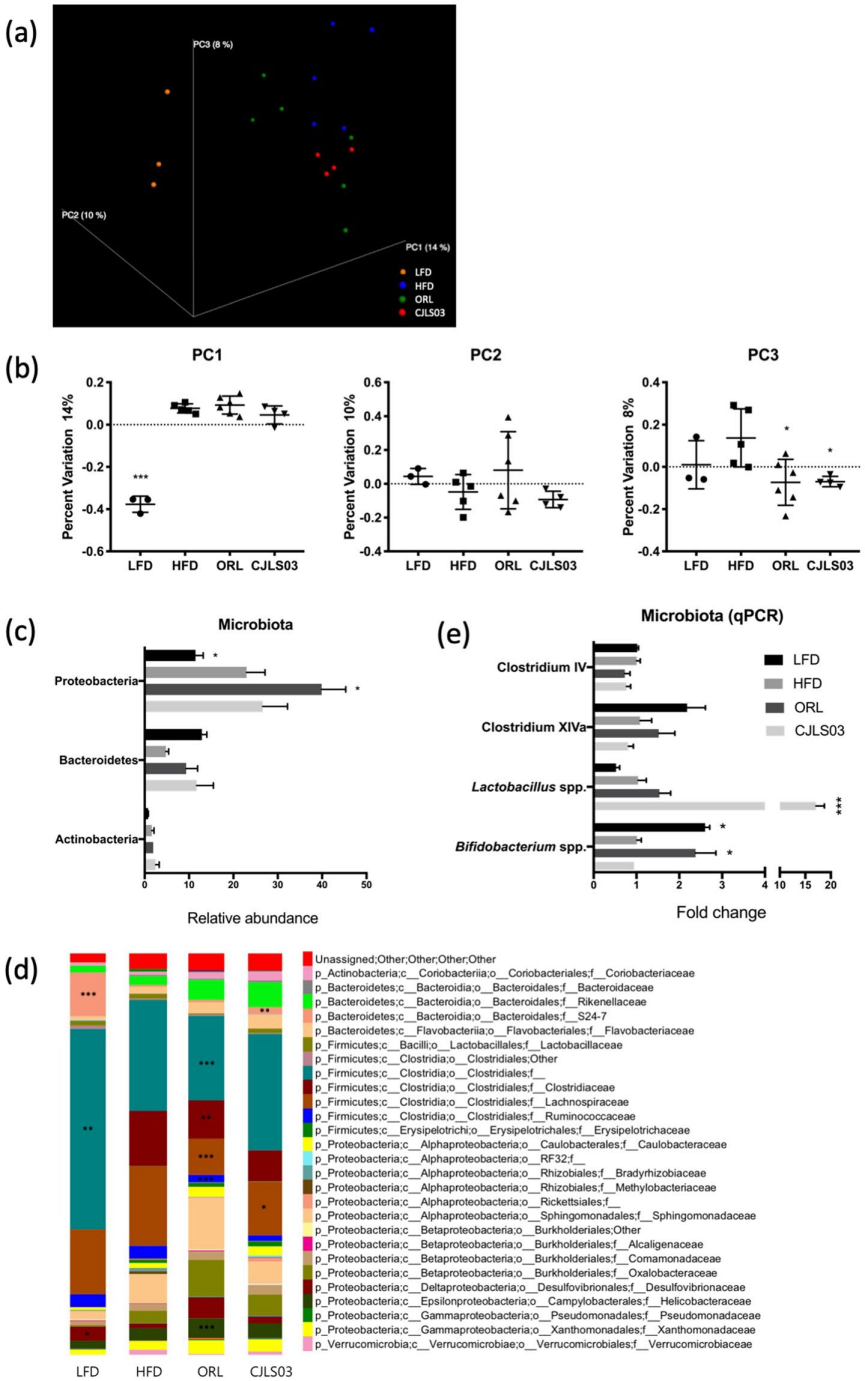
Microbiota analyses of faecal samples. Summary of taxonomic identification by a three dimensional PCoA plot of LFD, HFD, ORL and *L. sakei* CJLS03 treatment groups using unweighted unifrac (**a**), showing percentage variation (beta diversity) for each of PC1, PC2, PC3 (**b**), taxonomic summary of the phylum level (**c**), the family level (**d**), and some of the major groups/genera within the Firmicutes (the *Clostridium* clusters IV and XIVa and *Lactobacillus*) and the genus *Bifdobacterium* (Actinobacteria) as measured by qPCR (**e**). ANOVA, Dunnett’s multiple comparisons test was used to distinguish the level of significance based on probability of 0.05 (*), 0.01 (**) and <0.001 (***) compared to the HFD group. Feeding groups: LFD: low-fat diet plus PBS; HFD: high-fat diet and PBS; ORL: high-fat diet plus orlistat; CJLS03 high-fat diet plus *L. sakei* CJLS03.

The *L. sakei* CJLS03 feeding group showed significantly higher SCFA levels in the serum and faeces compared to the HFD control group and (only for the serum) the ORL group (Fig. 5a,c). Yet, total SCFA levels in the faecal samples were lower than for the LFD control and ORL groups. Levels of acetate and propionate were significantly higher in the serum of the *L. sakei* CJLS03 group than in the HFD and ORL groups, but with significantly higher butyrate levels in the LFD group. Significantly elevated levels only of acetate were detected in the faecal samples both of the *L. sakei* CJLS03 and LFD control feeding groups, while propionate and butyrate levels were significantly higher only in the LFD and ORL groups, when compared to the HFD and CJLS03 treatment (Fig. 5b,d). A noticeable taxonomic shift in the faecal microbiota was detected when using Qiime analysis (Fig. 6a). The PCoA plots strongly differed for all the high-fat diet feeding groups (beta diversity), with a higher percent variation towards PC1, while both Orlistat and *L. sakei* CJLS03 feeding induced a shift towards PC3 (Fig. 6b). *L. sakei* CJLS03 treatment showed no dramatic modulation of the major phyla, although, compared to the LFD, Orlistat resulted in a significant increase in the Proteobacteria (Fig. 6c). At the family level interesting diferences were detected when the HFD and both treatments (ORL and CJLS03) were compared with the LFD, with a strong reduction in the Bacteroidales Family S24-7, and increases in the *Rikenellaceae* (especially for the two treatments), the *Clostridiaceae*, the *Sphingomomonadaceae* and *Oxalobacteraceae* (Fig. 6d). Compared to the HFD control group, significantly elevated numbers of *Lactobacillus* spp. were detected in the CJLS03 group, while Orlistat treatment resulted in levels of *Bfidobacterium* spp. similar to those for the LFD control (Fig. 6e). Administration of *L. sakei* CJLS03 with the HFD had no effect on the Gram-positive groups *Clostridium* IV and XIVa (Firmicutes) and *Bifidobacterium* spp. (Actinobacteria) when compared to the HFD control, except for a highly significant (more than 15-fold) increase of the *Lactobacillus* population in the *L. sakei* CJLS03 group (Fig. 6e). On the other hand, Orlistat treatment with the HFD resulted in levels of *Bfidobacterium* spp. similar to those of the LFD control group (Fig. 6e).

## Discussion

We have investigated the anti-obesity effect of *L. sakei* CJLS03 using a diet-induced obese murine model and found that *L. sakei* CJLS03 administration resulted in a strong increase in SCFAs concomitantly with microbiota modulation. Compared to the HFD control group plus placebo the impact of this putative probiotic strain was comparable to that of the ORL group as was reflected in a significant weight and (epididymal and subcutaneous) adipose tissue reduction. Feed consumption of the LFD and ORL groups was significantly higher than the CJLS03 and HFD groups between which no changes could be detected. An overall probiotic derived anti-obesity effect was influenced by reduced fatty acid synthesis gene expression and up-regulated beta-oxidation in the EAT. A probiotic associated anti-obesity effect and related phenomena in HFD-fed mice were described by Wang and colleagues^7^. They consider probiotic induced glucose-insulin homeostasis in the EAT and microbiome modulation a major cause of weight gain attenuation. Yet, specific metabolites associated with and derived from the resident microbiota were not fully defined by these workers. Administration of *L. sakei* strain OK67 isolated from kimchi ameliorated HFD induced body and epididymal fat weight gains and reduced expression of various pro-inflammatory cytokines and serum endotoxin in a HFD induced obesity mouse model^9^. An earlier study has reported on the reduction of body weight, body mass index and visceral and subcutaneous fat by application of *L. gasseri* SBT2055 (in fermented milk) in a double-blind randomized, placebo-controlled trial over 12 weeks^10^. Increased numbers of indigestible carbohydrate utilizing microbiota, including members of the family *Ruminococcaceae* have been reported for obese individuals^18^. Since the caecal concentration of short chain fatty acids (SCFAs) produced by commensal microbial fermentation constitutes 10% of the daily absorbable energy, changes in the gastrointestinal tract (GIT) microbiota should therefore strongly impact host energy metabolism^19^. Faecal samples of overweight and obese individuals contained higher SCFA concentrations as compared to lean counterparts^20^. Riduara and colleagues reported that germ-free mice receiving (transplanted) faecal material of discordant obese and lean twins^21^, showed elevated levels of SCFAs in the caecum of those mice receiving faecal transplants of the lean twin concomitantly with a lean phenotype in the recipient mice^20^. Data from our study are pointing to SCFAs as possible anti-obesity metabolites of the (modulated) gut microbiota. In this study we have detected significantly elevated SCFA levels in faecal and serum samples of the *L. sakei* CJLS03 feeding group concomitantly with modulation of the microbiota. The role of butyrate in glucose-insulin homeostasis was described by Gao and colleagues^33^, showing the potential of dietary butyrate supplementation for prevention and treatment of diet-induced insulin resistance in mice. Butyrate has induced an increase in beta-cell proliferation and function in juvenile rats and also significantly decreased hyperglycemia in induced beta-cell apoptosis^34^, while it reduced insulin resistance in type-2 diabetic rats^35^. The underlying mechanism was proposed to be linked to energy expenditure and induction of mitochondria function and resulted from increased insulin expression^34^. Thus, direct feeding of butyrate resulted in reduced fat levels without reduction in food intake. Increased levels of AMPK activity and up-regulation of beta-oxidation genes were found in the butyrate feeding group, thus supporting the results recorded in this study. Moreover, it has been shown that a reduced lactate supply (e.g., by reduced lactic acid producing population) may lead to a depletion of butyrate producing taxa, resulting in a negative impact on the host immune system^36,37^. Not only butyrate but all major SCFAs, including acetate and propionate, have been reported to exert a beneficial role in host health, thereby providing a mechanistic basis for explaining the potential impact of microbiota modulation on host patho-physiological status also involving metabolic syndrome^27,38^. Den Besten and coworkers^39^ have shown the major three SCFAs (acetate, propionate and butyrate) to reduce lipogenesis and increase beta-oxidation in adipose tissue. Consequently, gut microbiota modulation resulting in SCFA increase may serve as an indication of a ‘healthy’ microbiota^23,40^. Still, SCFA production in the host gut is a highly complex process of interactive metabolic pathways, details of which are still mostly unveiled. As a lactic acid bacterium, *L. sakei* CJLS03 is not a butyrate producer *per se*; however, by producing lactate it can influence the gastrointestinal environment, modulate the gut microbial population (as, e.g., in the case of lactic acid bacteria such as enterococci)^41^, and provide a carbon source that can be further utilised for production of SCFAs^37^. This was reflected in the elevated levels of total SFCAs in the serum of the CJLS03 group (Fig. 4a), and suggested by the 15-fold higher abundance of *Lactobacillus* spp. in the CJLS03 group (Fig. 6e). Even when some lactic acid bacterial strains have been claimed to produce SCFAs (apart from acetate and lactate), increased SCFA concentrations have only been produced in interaction with complex gut microbial populations, e.g., in a simulated chicken caecum^42^, by the use of selected synbiotics in a model system of the human colon^43^, and in an *in vitro* (SHIME) simulator of the human gut^44,45^. Based on formerly published data, caecal microbiota may be stronger modulated by probiotic administration, thus resulting in a higher correlation of microbiota with major SCFA levels, typical of the distal small intestine and proximal large intestine^46^.

Compared to the HFD control, *L. sakei* CJLS03 feeding has influenced the host faecal microbiota in a different way than either the LFD control or the ORL treatment. Interestingly, the bifidobacteria of the ORL group (as detected by qRT-PCR) were “restored” to approximately the level of the LFD control, suggesting a beneficial effect of Orlistat on the *Bifidobacterium* population (but not the Actinobacteria as a whole) when administered to the group receiving the highfat diet (Fig. 6c and e). An assessment of the possible effects of Orlistat and other treatments on the *Bifidobacterium* population should, however, take into consideration the limitations given by the primers chosen to amplify the V1–V3 variable 16S rRNA regions. Specific sample preparation procedures include mechanical disruption (by bead-beating) for DNA extraction, and appear more reliable when applied in conjunction with “optimised universal PCR primers”^47^. Moreover, when using the MiSeq platform for sequencing of the V1–V3 region of the 16S rRNA gene the removing of low-frequency sequences may reduce error rates^48^. A specific ‘indicator’ bacterial group resulting from the *L. sakei* CJLS03 administration could not be detected in this study, except for a noticeable increase of the phylum *Bacteroidetes*, a significant increase of *Lactobacillus* species and also an increase in the abundance of the family *Rikenellaceae* (Fig. 6).

In conclusion, the administration of *L. sakei* CJLS03 in a diet-induced obese mouse model showed significant reduction of adipose tissue and an attenuation of various obesity associated biomarkers including serum cholesterol and triglyceride without reduction in feed consumption. These parameters were well correlated with increasing amounts of SCFAs, both in faecal and serum samples concomitantly with modulation of the gut microbiota. It has indeed been shown that obesity-enhancing changes in the mouse gut microbiota may be partially reversed by cessation of the high fat diet^49^. It appears that elevated SCFA levels may be an indication of a ‘healthier’ microbiota when compared to the HFD control group; this was most probably the result of an increased abundance in the gut (faecal) microbiota upon administration of strain CJLS03. Serum chemical analysis indicated no sign of toxicity while reduced free fatty acid and leptin levels were detected in the *L. sakei* CJLS03 feeding group. Overall, particular probiotics such as *L. sakei* CJLS03 may confer host health benefits by gut microbiota modulation and stimulation of SCFA production. The positive role of SCFAs as key bacterial metabolites has been recently highlighted in an extensive summary by Koh and colleagues^40^.

Changes in traditional lifestyle to that typical of industrialized countries seems to be associated with shifts in the gut microbiota^50^. *L. sakei* CJLS03 appears to be a promising probiotic candidate for combating the growing incidence of metabolic syndrome and obesity probably resulting from a shift in the gut microbiota. On the other hand, in underdeveloped countries, sufficient energy harvesting via the diet constitutes a major issue that may justify a different approach in probiotic development with a focus on the enhancement of energy conversion.

## Methods

All experiments were performed in accordance with relevant guidelines and regulations.

### Animal experiments

The animal study was approved by the ethical committee of Handong Global University, Pohang, South Korea. Four-week-old, specific pathogen free (SPF) male C57BL/6 mice were purchased from Saeron Bio, South Korea. High-fat diet (Research Diets D12492) (HFD), low-fat diet (Research diets D12450) (LFD) and composition of diet is described in Supplementary Table 1. Autoclaved tap water was provided *ad libitum*, while the animals were housed at 23 °C and 55 ± 10% humidity, in a 12 h light/dark cycle. All mice were separated into four different groups each receiving different treatments (Table 1).

**Table 1:** Study design and animal treatments based on a high-fat (HFD) and low-fat diet (LFD) in a murine model.

Group (n = 7)	Feed type	Treatment
LFD	LFD	200 μL PBS (non-obese control)
HFD	HFD	200 μL PBS (obese control)
ORL	HFD	Orlistat 40 mpk
CJLS03	HFD	1 × 10^9^ CFU/day of *L. sakei* CJLS03 suspended in 200 μL PBS

The experiment comprised three weeks of an adaptation period followed by eight weeks of oral administration of substrates listed in Table 1. On the last day of the experiment, the mice were sacrificed by dislocation of the cervical vertebrata. The organs, e.g., small intestine, liver, epididymal adipose tissue and mesenteric adipose tissue were collected, weighed, and kept at −80 °C until analysis. One part of adipose tissues of each group were kept in 10%v/v formalin/PBS, and then embedded in paraffin for staining with haematoxylin and eosin for microscopy. Adipocyte size measuring has been accepted as an indication of obesity. Average of three different randomly chosen adipocytes from five mice per each group was analysed to measure the size of adipocyte. Blood, acquired by cardiac puncture, was centrifuged for 20 min at 2500 g to isolate serum.

### Bacterial strains and culture conditions for the animal study

*L. sakei* CJLS03 was isolated from Korean fermented kimchi. This strain was grown in MRS (Difco Laboratories INC., USA) and prepared daily for feeding. It was grown for seven hours to reach late log phase and collected (16,000 *g*, 5 min, 4 °C) and washed twice with PBS. The strain was prepared in an approximate number of 1 × 10^9^ CFU/mL according to a pre-optimised standard curve (data not shown) using optical density by SPECTROstar Nano (BMG Labtech, USA). The prepared suspension (1 × 10^9^ CFU/mL) of the strain was suspended in 100 µL of PBS to be administered to each mouse by oral gavage daily.

### Extraction of microbial genomic DNA from faecal samples

Genomic DNA (gDNA) was extracted from mouse faeces using the ReliaPrep gDNA Tissue Miniprep System (Promega, USA), after mechanical disruption of microbial cell walls. Briefly, 50 mg of faecal samples were suspended in 720 μL of lysis buffer solution (320 μL of PBS + 400 μL of CLD) in a screw cap micro-tube (Sarstedt, Germany) together with 0.3 g of 0.1 mm zirconium/silica beads (Biospec, USA), followed by disruption in a mini-beadbeater-16 (Biospec, USA) for 2 min and centrifuged at 14,000 × g for 3 min at room temperature. The supernatant was thoroughly mixed with 250 μL of Binding Buffer (BBA) and placed on a binding column. For further cleaning and eluting the manufacturer’s instructions were followed.

### Gut microbiota analysis and bioinformatics

Gut microbial metagenome analysis was performed with the Roche 454 GS FLX plus system (AtoGen, Korea) using genomic DNA (gDNA). Briefly, primers were designed based on the V1–V3 variable region of 16S rDNA sequence (forward, 8f: 5′-AGAGTTTGATCMTGGCTCAG-3′; reverse 518r: 5′-ATTACCGCGGCTGCTGG-3′) and tagging of 10 bp unique barcode labels; the adaptor sequence was applied to monitor multiple samples in a single sequencing plate. Flow pattern B raw data from the 454 GS FLX plus system (Roche, Switzerland) was denoised and filtered using FlowClus^51^ and further analysed using MacQIIME^52^ 1.8.0 pipeline. Chimeras were eliminated using Usearch 6.1 and sequences were clustered into operational taxonomic units (OTU) at 99% sequence similarity, and taxonomically assigned using RDP database. Composition and inter-community beta diversity of taxonomically assigned microbiota were interpreted using three-dimensional (3D) principal coordinates analysis (PCoA) using unweighted UniFrac distance, analyzed and visualized by MacQIIME 1.9.1. Each PC1, PC2 and PC3 is presented separately as column scatter graph with mean and standard deviation, thereby showing the differences in taxonomic abundance profiles between the respective treatment groups (see Fig. 6b). Further clusters and species levels were measured using qRT-PCR as described by Ji *et al*.^13^, using specific primers for *Clostridium* cluster IV (forward-GCACAAGCAGTGGAGT, reverse-CTTCCTCCGTTTTGTCAA), *Clostridium* cluster XIVa (forward-AAATGACGGTACCTGACTAA, reverse-CTTTGAGTTTCATTCTTGCGAA), *Lactobacillus* species (forward-AGCAGTAGGGAATCTTCCA, reverse-CACCGCTACACATGGAG), and *Bifidobacterium* species (forward-GCGTGCTTAACACATGCAAGTC, reverse-CACCCGTTTCCAGGAGCTATT).

### Short chain fatty acid analysis

SCFA analysis was performed according to the method of Schwiertz *et al*.^20^. Briefly, 50 mg of deep-frozen faeces were mixed with 500 μL of extraction solution (comprising 0.1 mol oxalic acid /L and 40 mmol sodium azide /L), incubated in a horizontal shaker for an hour at room temperature, and centrifuged at 16000 × g for 10 min. 100ul of serum samples were prepared without the extraction step, but centrifuged briefly at 2000 g for 15 min without the extraction step. The supernatant was filtered through a 0.45 μm Minisart RC 4 syringe filter (Sartorius Stedim Biotech, Germany) and transferred to a Clear gas chromatography vial (Shimadzu, USA) and tightly sealed using Ribbed blue screw vial cap with bonded silicone (Shimadzu, USA) until analysis. A GC-2010 (Shimadzu, Japan) and HP-Innowax 30 m × 0.32 mm × 0.25 μm column (Agilent, USA) were used for detection; N_2_ gas served as carrier gas. 1 μL of each sample was injected by Shimadzu Auto-sampler AOC-20is (Shimadzu, Japan) at 260 °C and detected by flame ionised detector (FID). The column temperature was increased from 100 °C up to 180 °C at a rate of 25 °C/min. Volatile free acid standard mix (Supelco, USA) was used as analytical standard of C2 through C5.

### Blood analysis

50 µL of blood serum sample of each mouse were diluted into 450 µl of PBS and glucose, triglycerides, and cholesterol levels measured using an automated biochemical analyser BS-200 (Mindray, China) in Pohang Technopark (South Korea). The concentration of leptin in the serum was determined by the enzyme-linked immunosorbent assay (ELISA) method using ELISA Kit, pink-ONE (Koma, Korea) as described in the manual. Free fatty acids (FFA) were analysed using EnzyChrom Free Fatty Acid Assay Kit (BioAssay Systems, USA) according to the manufacturer’s manual.

### Measurement of host immune and obesity biomarkers

Extraction of mRNA from adipose tissue followed the specific protocol of RNeasy RNA tissue miniprep system (Promega, USA). Briefly, each organ sample was homogenised by a hand-held homogeniser (IKA, Germany) in lysis buffer and centrifuged. The supernatant was mixed with isopropanol and passed through a column provided with the kit. After several washing and DNase treatments, the purity and concentration of the eluted RNA were measured by SPECTROstar (BMG LABTECH, Germany). 2–3 µg of complement DNA (cDNA) were prepared using GoScript™ Reverse Transcription System (Promega, USA) through a Verity 96-well thermal cycler (ABI research, USA) after 10 min of incubation with oligodT primer at 70 °C. Various obesity related gene expression levels were monitored by the qRT-PCR method and calculated using the Delta Delta C(t) method normalised by GAPDH. Primer information is as follows: TNF-a (forward- ACTGCCAGAAGAGGCACTCC, reverse- CGATCACCCCGAAGTTCA), MCP-1 (forward - CGGAACCAAATGAGATCAGAA, reverse – TGTGGAAAAGGTAGTGGATGC), IL-1b (forward-GACCTTCCAGGATGAGGACA, reverse-AGCTCATATGGGTCCGACAG), IL-10 (forward-GGTTGCCAAGCCTTATCGGA, reverse-ACCTGCTCCACTGCCTTGCT), SREBP-1c (forward-AGCAGCCCCTAGAACAAACAC, reverse-CAGCAGTGAGTCTGCCTTGAT), FAS (forward- CTGGACTCGCTCATGGGTG, reverse-CATTTCCTGAAGTTTCCGCAG), SCD1 (forward-TCAACTTCACCACGTTCTTCA, reverse-CTCCCGTCTCCAGTTCTCTT), PPAR-a (forward-GTACGGTGTGTATGAAGCCATCTT, reverse-GCCGTACGCGATCAGCAT), CPT1 (forward-AAGCCTTTGGGTGGATATGTGA, reverse-ATGGAACTGGTGGCCAATGA), UCP2 (forward-CTACAAGACCATTGCACGAGAGG, reverse-AGCTGCTCATAGGTGACAAACAT). Proteins were extracted from EAT using PRO-PREP buffer (iNtRON Biotechnology Inc., Korea). Samples were analysed by SDS-PAGE-immunoblotting assay. Antibodies (Cell signalling technology, Beverly, MA) against phospho-AMPK (Thr172), total AMPK and GAPDH were used as primary antibodies, followed by anti-rabbit IgG-HRP conjugated secondary antibody.

### Statistical analysis

All values were expressed in average and standard deviation (SD) and experimental determinants were confirmed with at least triplicate biological samples. ANOVA, Dunnett’s multiple comparisons test was used to distinguish the level of significance based on probability of 0.033 (*), 0.002 (**) and <0.001 (***).

## Supplementary information

Supplementary information

## Data Availability

The datasets generated during and/or analysed during the current study are available from the corresponding author on reasonable request.
