# Supplementary material for: Amelioration of obesity-related biomarkers by Lactobacillus sakei CJLS03 in a high-fat diet-induced obese murine model
Source: Sci Rep. 2019 May 2;9:6821. doi: 10.1038/s41598-019-43092-y (PMC6497927; doi:10.1038/s41598-019-43092-y)
Supplement: Supplementary file 1 — Supplementary information [file 41598_2019_43092_MOESM1_ESM.pdf]

Supplementary information

Amelioration of obesity-related features by *Lactobacillus sakei* CJLS03 in a high-fat diet-induced obese murine model

Yosep Ji[1], Soyoung Park[1], Youngmee Chung[2], Bobae Kim[1,4], Haryung Park[1], Eunchong Huang[1], Dahye Jeong[2], Hoe-Yune Jung[3], Bongjoon Kim[2], Chang-Kee Hyun[1,4], Wilhelm H. Holzapfel[1*]

[1]Advanced Green Energy and Environment, Handong Global University, Pohang, Gyungbuk 37554, Republic of Korea;

[2]CJ Blossom Park, 42, Gwanggyo-ro, Yeongtong-gu, Suwon-si, Gyeonggi-do, 16495, Republic of Korea;

[3]Department of Life Science, Division of Integrative Biosciences and Biotechnology, POSTECH, Pohang, Gyungbuk 37673, Republic of Korea;

[4]School of Life Sciences, Handong Global University, Pohang, Gyungbuk 37554, Republic of Korea

*Correspondence and requests for materials should be addressed to W.H.H. (email: wilhelm@woodapple.net)

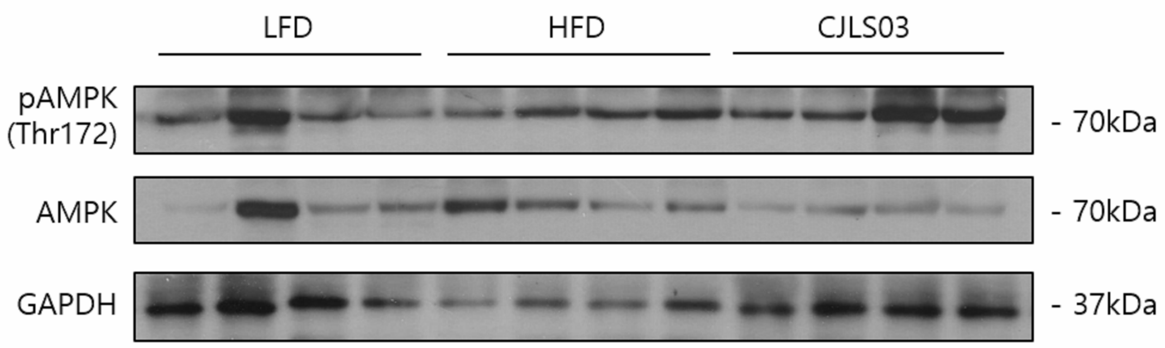

Supplementary Figure S1. Western blot of 5′ AMP-activated protein kinase (AMPK) and phosphorylated AMPKα (Thr 172) as host obesity and immune associated biomarkers in epididymal adipose tissue after L. sakei CJLP03 application in the diet-induced obese murine model. For explanation of treatment and abbreviations, see Table 1.

Supplementary Table 1. Composition of high-fat and low-fat (control) diets.

	High-fat diet (5.24 kcal/g)		Control diet (3.85 kcal/g)	
	g %	kcal %	g %	kcal %
Protein	26.2	20	19.2	20
Carbohydrate	26.3	20s	67.3	70
Fat	34.9	60	4.3	10
	g	kcal	g	kcal
Casein 80-mesh	200	800	200	800
L-Cystine	3	12	3	12
Cornstarch	0	0	315	1260
Maltodextrin 10	125	500	35	140
Sucrose	68.8	275.2	350	1400
Cellulose BW200	50	0	50	0
Soybean oil	25	225	25	225
Lard	245	2205	20	180
Mineral mix S10026	10	0	10	0
Dicalcium phosphate	13	0	13	0
Calcium carbonate	5.5	0	5.5	0
Potassium citrate	16.5	0	16.5	0
Vitamin mix V10001	10	40	10	40
Choline bitartrate	2	0	2	0
FD and C dye	0.05	0	0.05	0
